# The Challenging Process of Developing an Antenatal Social Intervention for Parents From Culturally Diverse Backgrounds to Reduce Postnatal Distress: A Participatory Action Research Study

**DOI:** 10.1111/hex.70135

**Published:** 2025-01-06

**Authors:** Sophie Isobel, Kahala Dixon, Alison Tutt, Bridget Clay, Sylvia Lim‐Gibson

**Affiliations:** ^1^ University of Sydney, Faculty of Medicine and Health Camperdown New South Wales Australia; ^2^ Department of Mental Health Sydney Local Health District Camperdown New South Wales Australia

**Keywords:** co‐design, cultural diversity, midwifery, participatory action research, perinatal mental health

## Abstract

**Background:**

A lack of social support contributes to women from culturally diverse backgrounds experiencing higher rates of perinatal distress and lower rates of service engagement.

**Objective/Methods:**

This participatory action research study aimed to understand what a culturally appropriate social intervention may look like for pregnant women from culturally diverse backgrounds. Field notes and qualitative transcripts were descriptively synthesised.

**Results:**

Challenges of engaging with culturally diverse communities in the context of perinatal health services were identified. Cultural factors and practices were seen to impact upon service engagement, with parents more likely to seek support outside of health settings. Community members expressed frustrations with the lack of deep cultural sensitivity in the structure and delivery of health services. Clear definitions in scope and aim of any intervention were indicated, before further community engagement.

**Discussion:**

Challenges in engaging individuals and services from diverse communities highlighted the risks of ideas embedded in oversimplified understandings based on cultural stereotypes and assumptions of homogeneity of experiences at the intersection of cultural diversity, perinatal distress and health services.

**Conclusions:**

Deep cultural sensitivity requires an understanding of how members of population groups perceive and understand health and wellbeing to directly inform the development of any intervention. Attempting to design a culturally sensitive intervention for socially isolated and culturally diverse parents within mainstream health services, led to a paradoxical tension between attempting to address needs in culturally insensitive ways or not attempting to address the needs at all.

**Patient or Public Contribution:**

Members of the public and people who identified as having lived experience of social isolation, cultural diversity or mental distress were engaged in the community consultation phase of the study.

## 1 Introduction

Addressing the perinatal mental health needs of women from culturally diverse backgrounds is a public health priority [1, 2, 3]. Women from culturally diverse backgrounds are known to experience increased rates of perinatal depression or distress [4, 5] and increased suicidal ideation [6]. However, they are also less likely to engage with health services [7, 8] due to sociocultural, structural and service barriers [5].

While some risk factors for perinatal depression are universal, social factors also play a critical role [9]. For culturally diverse parents, traditional support networks are often missing, approaches to child‐rearing or birthing may not be recognised and a lack of culturally acceptable information may be supplied [10]. Lack of social support is known to play a role in the development and maintenance of perinatal distress for women from culturally diverse backgrounds [9, 11, 12, 13, 14] and may contribute to adverse maternal and child outcomes [15]. This suggests a need for further understanding of what culturally appropriate integrated interventions may facilitate the development of social support, to buffer against perinatal depression or distress [9].

Currently in Australia, universal psychosocial screening occurs for all people birthing in public hospitals. Pregnant people who report distress or isolation may be allocated to clinicians based in the hospital or flagged on discharge with community child and family health services. While these are important clinical mechanisms for responding to distress or vulnerability, they are not focused on addressing wider issues of social and community isolation and displacement. Culturally diverse women in Australia have described isolation, loneliness, fear and anxiety in the perinatal period, compounded by poor social supports and inability to uphold cultural practices that usually would involve their mothers and extended family across the perinatal period, alongside an expressed desire for culturally appropriate support groups where they can access information and interact with other mothers [12]. Socially focused groups in the perinatal period can reduce the incidence of postnatal depression for culturally diverse parents when they are developed with social validity and cultural sensitivity [9]. Preventative approaches are needed to minimise ongoing need for services, reducing intergenerational challenges, distress and service costs [16]; while improving health outcomes for diverse communities [17].

There are known challenges in developing culturally safe and sensitive interventions within mainstream health services. A western paradigm dominates the design and delivery of healthcare in Australia, with a focus on reaction rather than prevention and western conceptualisations of health and wellbeing that negate many of the social, cultural, economic, spiritual and political drivers of ill‐health [18]. This is amplified in perinatal services through biomedical approaches to birth, despite increasing attention to continuity and community‐based models of midwifery‐led care and widespread discourses of cultural sensitivity. Cultural sensitivity refers to the extent that the design, delivery and evaluation of health programmes incorporate the cultural experiences, values, expectations and beliefs of populations, in context of historical, environmental and social forces. Resnicow et al. [19] identify that cultural sensitivity has both surface and deep dimensions. The surface dimension refers to the superficial ‘matching’ of an intervention to a group to enhance acceptability. The deeper dimension requires understanding of the cultural, social, historical, environmental and psychological forces which influence health, wellbeing and health behaviour amongst populations. Both are important as surface sensitivity is required for feasibility, while deep sensitivity is required for efficacy and impact [19].

There is clear identification of the need for socially focused interventions for culturally diverse parents and increased sensitivity to cultural issues within perinatal care in the literature. However, far less is documented about specific interventions or approaches. This project emerged from within a health service, where a need was identified by clinicians to better address the social wellbeing of pregnant women from culturally diverse backgrounds. Local seed funding was received to develop ‘an intervention’. The local area within Sydney, Australia, has one of the most culturally diverse populations in the state and one of the highest birth rates. Within the local hospital, parents access midwifery and obstetric care and are referred to community‐based child and family health services in the postnatal period for early parenting support. This local area provides an appropriate setting for an integrated service collaboration to develop a culturally sensitive intervention to promote social connection for culturally diverse parents.

## 2 Aim

This project was based on a hypothesis that increasing social connectivity in a culturally sensitive way during pregnancy may prevent postnatal distress in a population of women from culturally diverse backgrounds. However, developing effective and culturally sensitive interventions requires consultation and co‐design.

The project aimed to facilitate increased understanding of the needs of culturally diverse parents within the identified community, for the purpose of developing a preventative intervention delivered within existing service structures. While the proposed intervention is hoped to increase antenatal social connection, confidence and wellbeing amongst parents, to reduce postnatal distress, the aim of this article is to describe the process of developing the intervention through consultation and participatory action. The research question for this process was ‘Is a health‐service‐based social intervention for women from culturally diverse backgrounds appropriate for reducing perinatal distress from the perspectives of health and community representatives?’

## 3 Method

A participatory action research (PAR) methodology was used. PAR is an interpretivist method. That is, it focuses on the complexity of human sense‐making within situations [20]. It is an iterative research approach to research that involves the populations being researched as researchers and blends theory and practice through action, evaluation and critical reflection [21, 22]. The purpose of PAR is to bring about change in specific contexts. Meyer [23] maintains that PAR's strength lies in its focus on generating solutions to practical problems and its ability to empower practitioners by getting them to engage with research and subsequent activities. PAR is a continuous learning process where researchers learn and share newly generated knowledge with those who may benefit or be impacted from it, while allowing practitioners to acquire information by practical application of solutions to specific problems in their work [24]. This paper reports on the development phase of the intervention as this is a key part of the process of PAR, and an underreported aspect of clinical research.

Initially, an advisory group (AG) was established with representatives from across local health services. Key services were invited to nominate an interested member for the AG. The AG were professionals from services who work with parents from culturally diverse backgrounds. The AG was intended to scope the project as well as identity community stakeholder representatives. The AG advised on the service needs of families in the local area, existing services and programmes, and reviewed the emergent intervention in relation to existing service delivery and the observed needs of the community. Essentially the AG provided guidance on the surface cultural sensitivity of the planned intervention. The AG met monthly for 3 months, all meetings were recorded and transcribed. Subsequently, a break of 3 months occurred to enable community consultation.

The work of the project was undertaken by a working group who met between sessions and progressed the ideas. Following three AG meetings, community stakeholders were identified. Individual consultation sessions were undertaken with community stakeholders by members of the working group to identify needs and determine what an effective culturally safe intervention could look like. The community consultation was intended to guide the development of the deeper cultural sensitivity of the proposed intervention. Community consultation occurred between August and October 2023. Consultation sessions were conducted individually via telephone or videoconferencing and detailed notes taken.

Community consultation focused on how perinatal care, wellbeing and health are perceived within the culturally diverse community and the cultural acceptability of a group intervention. As guided by [19], this period of consultation also sought to elucidate how the community may perceive that social isolation and perinatal distress may be different in their community to mainstream Australian society. Following community consultation, further consultation was undertaken within key services to check how ideas would fit within existing care delivery and to ensure an integrated approach. Findings were then returned to the AG for further refinement.

Ethics approval was gained from the Royal Prince Alfred Hospital Human Research Ethics Committee. Ethical conduct in PAR has unique challenges, particularly related to power relations and disruptions to the ‘usual processes’ of research [25, 26]. In this project, ethical conduct was considered as an ongoing process, in line with what Banks et al. [25], refer to as ‘everyday ethics’. Because of the blurred delineation between usual work practice and research, a transparent approach was prioritised where all participating or potentially participating individuals were sent written information about the project and the collection of research data before any meeting or consultation, and were also reminded at the commencement of each session that data was being collected and how it would be used. To manage expectations, it was essential to ensure participants were aware that the PAR process meant that the outcome of the project was not certain (i.e. a group may not occur) and the timeline may be slower than anticipated. All participants participated within their paid work hours, either within health or within community organisations and were given an opportunity to opt out of their data being included in the research. All meetings were transcribed or detailed notes taken.

Thus, study data included meeting minutes, field notes and project documents. All data were analysed using an inductive content analysis approach. This approach involves constant comparisons between qualitative data sources to iteratively organise and synthesise their content [27]. While similar to other thematic and qualitative approaches, inductive content analysis is most appropriate for small research activities with direct application to healthcare practice [27].

## 4 Findings

### 4.1 Project Development: Initial Advisory Group Meetings

Three initial AG meetings were held monthly between June‐August 2023. The AG included representatives from Diversity Services, Population Health, Child and Family Health, Maternity, Psychiatry and Perinatal Infant Mental Health Services.

Initial discussions identified several challenges of engagement with culturally and linguistically diverse (CALD) communities in the perinatal context. Building relationships with outreach community groups was highlighted as crucial for engagement in any intervention, while hospital midwives were identified as essential for identifying possible parents. It was noted that there may be cultural factors that delay initial engagement with child and family health services, and a postnatal intervention, such as the need to adhere to certain post‐birth practices within communities.

The AG identified specific prevalent local CALD communities and suggested collaborating with existing community groups and other local organisations. To enhance engagement, community settings from an intervention were recommended over hospital‐based locations. Participants identified CALD community resistance to engaging with health services unless accompanied by a cultural support worker. The need for a free intervention was emphasised, as some individuals may not have universal healthcare and be nervous of possible costs.

Cultural considerations were discussed, such as not running programmes during cultural festival periods, challenges with public transport, and the need for consistency of group facilitators to faciliate trust. The role of interpreters was highlighted as crucial in facilitating effective communication. The AG advisory group stressed the need for clear definitions in the study, such as migrant recency period, mental health, and timeframe of engagement, to ensure accuracy and relevance.

The AG provided guidance on the focus of the group:

A lot of the women coming to [the hospital] are really well educated in their own culture But they often aren't very health literate in ours, which is completely and utterly understandable… I would imagine that basic health literacy and system literacy…would be a very big part of what you'd be trying to do.

(Child and Family Health)

While also guiding flexibility and consideration of assumptions:

[you] said that the aim was prevention and wellness. And I just sort of thought about that concept of wellness and what that actually means. Like if you actually try and define health, it's actually quite a difficult concept to define…it's not simply the absence of disease or disorder. So I guess what I'm getting at is considering what the definition of wellness and health is for each of these different cultures, and it might be different. Rather than going in with sort of a single uniform concept of sort of what we're trying to aim for.

(Psychiatry)

In discussing how to focus the group, AG participants had differing ideas about whether to keep the cultural focus broad, mono‐cultural or targeted. Examples included

you may have populations that are at war with one another.

(Psychiatry)

and

If we're trying to promote social engagement, I don't know if [mixing cultural groups] will achieve that cohesiveness…. you might have a Vietnamese group who are really happy to meet at a cafe and park and walk their prams later and then you've got a group of Bengali women who won't do that if their husbands aren't with them.

(Midwifery)

At the second AG meeting, the conversation focused more specifically on some of the details that required refinement. For example:

There are kind of two things we're trying to do at once… think about ‘the what’ and think about ‘the who’. And we went down this rabbit hole of getting more and more specific with ‘the who’ but now we've come back to a place of understanding this as a pilot. Perhaps we need to be more inclusive and talk about… ‘new to Australia’ being an elusive idea. You can be new because you arrived months or weeks ago or you can feel new even though you've lived here for years and so it's less of a ‘this is who we think this group should fit’, but an opportunity for us to see how it might fit.

(Facilitator)

At this meeting, decisions were made to focus on a 4‐session group for women who were willing and able to attend a group at a nearby community venue. A structure of 3 antenatal sessions and 1 postnatal session was devised, planned facilitation by midwifery, child and family health and mental health clinicians. Recency of migration was recognised as hard to define and a loose definition was adopted as shared experience was seen as part of social connection. For example:

In terms of recency and engagement, I wouldn't be inclined to narrow it to like only ‘arrived in the last year’. The benefit of that is if you've got a Bengali woman who's been here for 5 months and a Bengali woman who's been here for 5 years, the one who's been here for 5 years can actually help the woman who's been here for 5 months navigate some of that local knowledge that you just don't know until you live here for a while. So, I wouldn't see a benefit to making that scope too narrow.

(Midwifery)

The AG were supportive of the draft outline being taken to wider community consultation to better understand how the group may fit with the needs of the community. As detailed here:

I think for me, the first one of the questions I would look to answering is… I know that there are some programs already in existence…. why are women not attending those? I think that's really the first question, to then be able to sort of identify… what's the actual gap.

(Population Health)

### 4.2 Cultural Sensitivity Refinement: Community Consultation

Community consultation occurred with representatives from local organisations who provide existing services to culturally diverse parents. Organisations included Local Council community development and cultural officers, local playgroups, organisations providing culturally specific services and multicultural services. Community consultation identified that there are a variety of existing cultural groups run in the community that have high rates of attendance by diverse parents yet it was difficult to elucidate what the contributing factors were to the success of these groups. Community participants described a need for informal engagement and less didactic content to allow space and safety for participants to identify their own needs and form social connections.

Participants highlighted a need to not replicate existing services or repeat information that families already receive elsewhere and instead open space for considering how culture may intersect with this information, for example, the structure of perinatal services or expectations of birth. Participants reflected that advice or information commonly shared during this period may not account for deeply held beliefs about parenting or family, for example, co‐sleeping, what to feed babies or parental roles in providing care, with some families finding the policies of health services to be contradictory to their beliefs. One council worker described observing how parents would often ask parenting questions during ‘playtime in the park’ that they would never ask at more formal sessions in venues or health centres. Participants described the agency of many families in seeking out information that aligned to their cultural beliefs. For example, one cultural worker described driving long distances to connect with other parents from her culture, rather than attending groups that were nonspecific. Community members expressed frustrations with ethnocentric approaches in health services but also had a lack of clarity about what gaps exist in the community or what form an intervention should take. Participants highlighted examples of ‘failed ideas’ such as a piloted multi‐cultural fathers' group and a young parent's mono‐cultural group, as well as ‘successful ideas’ such as longstanding and well‐attended playgroups; however, distinguishing features weren't clear. Having community involvement in any intervention was seen as crucial as some groups would need to be culturally ‘ushered in’ by members of community.

The need to consider the migration history of groups and how this can influence experiences was also discussed, with details of visa status and experiences of settlement impacting people's connection to the area and to each other. Participants acknowledged the difficulties in ‘choosing’ a population as the local area is comprised of so many diverse groups who all could benefit from support. Participants also could not identify a specific unmet need.

Community consultation highlighted the need for any intervention to consider the role of power and historical events when defining the included populations, referencing examples of groups who may feel very uncomfortable in a group setting together. Community members also noted that in some cultures groups themselves are not acceptable forums.

### 4.3 Intervention Refinement: Working Group

The working group identified that there were no clear answers to many of the questions that were asked throughout the consultation stages. A lack of clarity about the focus of the intervention and difficulty contacting key community groups resulted in a lack of rich data about the cultural acceptability of the proposal. Across services and individuals, conflicting information was given at nearly every point‐ about whether the group should be based in the community or hospital; whether partners should attend or not; whether to focus on one cultural group or many; whether there was a great need or a great reluctance for attending; whether content should be structured or unstructured and so on. As a result, pragmatic decisions were necessary. It became clear that there was no one way to serve the community and that diversity inherent to the culturally diverse community was a barrier to interventions being successful. Rather than designing the ‘perfect’ intervention, the working group decided to focus on piloting a social intervention within existing service structures to address perceived gaps in local service provision, rather than assumed community needs. Decisions were made that group focus would be refined based on engagement and interest and that practical decisions about the use of interpreters or cultural support workers would be decided based on who enrolled.

The working group determined that it was important to fit the intervention within existing resources and acknowledge service and cultural limitations but not be stopped by them. Decisions were made that the group would be semi‐structured but not prescriptive, didactic or educational (see Table 1). While the intervention would target social outcomes, the working group also recognised the need to acknowledge the wider context in which these occur, including a reluctance to engage with people from other cultures, conflicting cultural norms and cultural expectations of new parents.

### 4.4 Proposed Intervention

**Table 1:** Outline of proposed intervention.

Session 1	**Theme:** Keeping well during pregnancy (physical, emotional and social) **Objectives:** To engage participants in discussion on the importance of antenatal physical health; To engage participants in discussion on the importance of antenatal emotional health; To explore understandings of health behaviours and practices during pregnancy **Aims:** Introduce group and start to build connections Empower participants to take proactive steps in maintaining their physical and emotional health Foster a supportive environment for sharing experiences and concerns
Session 2	**Theme:** Accessing services and supports during the perinatal period **Objectives:** Support participants with knowledge about local resources and services available to them; Promote networking and community building **Aims:** Ensure that participants are aware of the support systems they can access. Foster a sense of community among participants.
Session 3	**Theme:** Sustaining connections during birth and postnatal period **Objectives:** Explore perceptions and beliefs about birthing options and postnatal care; Provide an opportunity to discuss common concerns about childbirth and postpartum; share cultural traditions around birth and recovery **Aims:** Create a safe space for discussing childbirth and postpartum challenges
Session 4	**Theme:** life with a baby and beyond **Objectives:** Provide opportunities for participants to share about their experiences of early parenting; Explore ideas of how to stay well postnatally (both mental health and physical wellbeing) **Aims:** Support participants to feel more emotionally equipped for life with a baby; Promote self‐care, ongoing social connection and mental well‐being

To ensure feasibility of the pilot within existing service provision, the need for further consultation with midwifery and child and family health services was identified as these services will be identifying parents, enrolling them in the programme and providing co‐facilitation.

### 4.5 Intervention Refinement: Internal Consultation

Further internal midwifery and child and family health consultation was sought to identify whether the proposed intervention and recruitment were feasible. Consultation identified a number of initiatives within existing perinatal settings which have attempted to engage specific cultural groups, for example, Arabic gestational diabetes education sessions or Bengali new parents' groups. However, a potential gap was identified in the delivery of a co‐facilitated group with a flexible focus on social connection and engagement in existing care pathways across the perinatal period.

Practical challenges were raised. For example, many socially isolated parents are dependent upon their partners to drive them to appointments. Midwifery staff identified existing difficulties in encouraging socially isolated CALD parents to attend essential appointments for medical concerns and that getting parents to attend additional optional opportunities may be difficult. Many parents were also identified to rarely attend existing antenatal classes due to transport, childcare and cultural reasons. Midwives described attempting to run numerous specific language and cultural groups during the antenatal period but not finding that targeting specific groups improved engagement. They also reported that mixed cultural groups did not improve uptake. While incentives were considered, an example was also given of a research project which sought to recruit parents with financial incentives for participation, yet engagement was still minimal. Video conferencing alternatives had also been attempted due to transport and childcare issues, with no engagement. Despite this, midwifery services were open to supporting a pilot project to attempt to engage CALD parents as they strongly identified a desire and need to ensure social connection for parents who may not access existing services. Similarly, child and family health services described feeling unsure how to better engage culturally diverse or recently migrated families in their services but expressed a keenness to work collaboratively to pilot an intervention. Additional benefits were proposed of improved relationships between the antenatal and postnatal care providers who would be working with these parents. The flexible outline of the proposed intervention was endorsed by the stakeholders.

### 4.6 Finalising Plans: Further Advisory Group Meetings

A final first‐stage AG meeting was held in November 2023, where the research team provided feedback on the community consultation, presented the draft outline and discussed the challenges in incorporating all feedback in ways that were feasible. As described by a facilitator:

I imagine that you're all sitting there going: “Yes, we could have told you that there's no magic answer of a thing that hasn't been tried that could fix the issue of a lack of social connection”….[in our consultation] we've also discovered that many innovative things have been attempted. There's lots of existing resources in the communities and within your services…and so how we get people linked into those rather than replicate those has been something we've been trying to nut out as well. We hit a definite stumbling block around that. Groups are not always culturally appropriate and at that point I think we were ready to just quit… we can have a surface cultural sensitivity of being really inclusive and appropriate with our language and offering something, but how we can deeply offer something that aligns to cultural values is challenging within health systems and services. Yet, at the same time as… we were hearing a lot of challenges, we were also hearing a lot of really high expectations and hopes for what is possible…so… the consultation has given us a lot of information and at times felt quite overwhelming and impossible, and at other times felt excellent and full of possibility and at some point, we've had to just make some decisions and go OK we need to pilot a thing and we don't wanna be put off by the fact that it's hard and therefore we shouldn't do anything.

(Facilitator)

Despite the challenges the AG were keen to continue with the pilot of the group to gauge interest from the target population. The AG were all supportive of keeping a service‐specific focus and seeing what was possible in engaging parents. The risk of deciding it was ‘too hard’ was seen as undesirable, despite the challenges. Discussion subsequently focussed on gathering data about the amount of parents referred to the intervention, and the amount who enrol and attend, and where possible seeking qualitative feedback from parents about how the pilot could better addressed their social needs. One member described

I think anyone who's worked in the local district… understands all of what you've found and it it's kind of both reassuring and still overwhelming to hear that…And I think that just leaping off and giving this a try with a kind of diverse group and seeing how that rolls is a great thing because I just think you're not gonna satisfy everyone's sensibilities every time. And really, you're trying to, you know, identify women who would benefit. And there will be heaps who will, and it's just about how do we actually, in each group, cater to the needs of those women and be sensitive to their particular situations. And I just think it's a good decision just to offer it to any woman who is a new arrival from wherever and just see how it pans out. And then tweak.

(Population Health)

Subsequently, the design phase of the project was completed with a programme for a proposed social group intervention, alongside ongoing uncertainty about its cultural sensitivity or applicability, but a willingness from existing services to trial the intervention.

**Figure 1:**
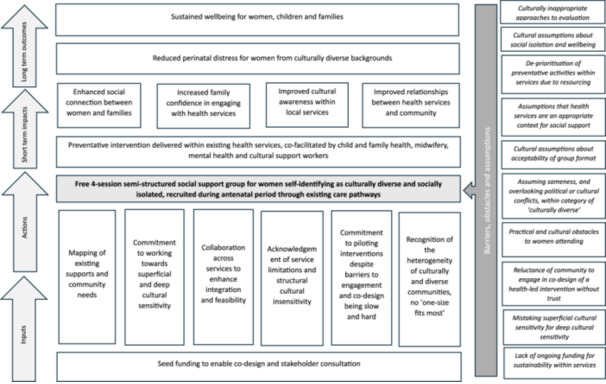
Program logic.

Iterative refinement of the intervention will occur as part of the pilot. To capture the outputs from the PAR process, a logic model was generated to capture the theory of change of the proposed intervention (see Figure 1). Logic models are one way of showing how activities are anticipated to achieve their intended outcomes [28].

## 5 Discussion

This participatory action research study captures the process of designing an intervention to meet the assumed needs of a population identified by health services as vulnerable. The intersection of cultural diversity, perinatal distress and health services highlighted the heterogenous nature of these experiences and a paradoxical tension between attempting to address needs in culturally insensitive ways or not attempting to address needs at all. Interventions to provide culturally appropriate maternity services are by nature context‐specific [29] and as such a PAR design for developing the intervention was indicated and beneficial, with the health and community consultations providing local and detailed feedback on the context of care for families. Documenting the process and not just the outcome is a strength of PAR approaches.

In this study, through documenting the staged process of consultation and iterative refinement, the findings highlight both the complexity of seeking consensus and clarity in a multistakeholder project within complex health services, and the challenges of attempting to address the needs of a diverse population identified as vulnerable and not accessing services, from within those services.

The design phase of this project identified challenges associated with cultural sensitivity and inclusivity and a lack of cultural safety within many mainstream health service approaches, despite significant attempts and intent by clinical teams. Deep cultural sensitivity requires understanding of how members of population groups perceive and understand health and wellbeing, such that beliefs and core cultural values directly inform the development of the intervention [19]. It was not possible to determine the deep cultural sensitivity of the proposed intervention due to the heterogenous nature of groups described as culturally diverse and a lack of co‐design of the initial problem statement. Focusing on CALD populations broadly is known to promote oversimplified understandings based on cultural stereotypes and a tendency to homogenise diverse people into a collective ‘they’ [30].

Documented culturally sensitive interventions during the perinatal period are limited in number and detailed description [31]. Many attempts at adapting interventions to be culturally sensitive focus on superficial characteristics of target populations [31], often based on practitioners' perceptions of diverse community needs [32], as occurred in this study when attempts to identify a venue or a focus were determined by assumptions of what groups may want. Community participation is crucial for understanding problems and solutions from the community perspective [33]. Yet, while there was significant stakeholder engagement in this project, there was no formal lived experience engagement. Many of the community stakeholders identified as members of the target population, but they were representing their professional roles in participation, rather than providing lived experience perspectives. While it was hoped that CALD parents may also participate in the consultation, this was impeded by the challenges in engaging with organisations connected to culturally diverse families in the community. This is a critical gap in the development of the intervention.

The perinatal period is a social and cultural event governed by norms. Dominant cultures are expressed through social institutions which then influence how health issues are defined, assessed and responded to. Differences between services and service users' understandings are a major issue in service delivery, with cultural insensitivity and misattunement known to result in a lack of trust in services and service providers [34]. Developing culturally sensitive interventions thus requires close collaboration with the identified communities as collaborators and experts [31] to ensure that the initial focus is inclusive and appropriate. Otherwise, attempts at cultural sensitivity can obscure rather than facilitate cultural sensitivity through stereotypes [35]. Through the community consultation phase of this study, participants described that many people from culturally diverse backgrounds disengage with health services without explanation due to cultural mismatch and insensitivity of approach. Similarly, without co‐design of the initial focus of this project, it is possible that disengagement from the refinement and delivery of the intervention would be a marker of a deeper mismatch of cultural assumptions about what it means to be socially isolated, recently migrated, experiencing perinatal distress or the relevance or acceptability of group interventions. This is also linked to the many cultural subgroups with differing languages, experiences and levels of acculturation within the population identified broadly as culturally and linguistically diverse [32].

How to deliver culturally appropriate integrated interventions is complex [34] as cultural factors are often not the only factors affecting peoples' use of perinatal services. Social, economic, geographical and political factors also intersect to impact engagement and access [29]. Interventions are also embedded in cultural assumptions concerning the nature of individuals, relationships, interactions, health and recovery, usually embedded in westernised worldviews of individuality, identity and control [36]. Cultural safety in care occurs through being aware of difference, power relations, implementing reflective practice, and by service users determining what encounters they experience as safe [30]. Cultural safety therefore requires clinicians and researchers to examine the potential impact of their own biases, attitudes, assumptions, stereotypes and prejudices and active reflection on the culture of the service, rather than the culture of the diverse individual [30]. In this study, rather than seeking further input from the community about their experiences of social isolation, a pivot occurred midway through the consultation phase to focussing more closely on the context of care provision across maternity and child and family health services to determine what gaps exist within care. The advisory group provided a forum for reflecting on the assumptions that underpinned the approach, however the cultural safety of the intervention can only be determined through the actions and feedback of the parents who are referred.

Co‐design has become a buzzword across sectors but can face significant challenges when attempted in real‐world service settings. Challenges include that the process may take more resources and time than expected, may encounter a reluctance to engage participants despite best intentions, or may discourage the researchers or co‐design team from continuing their work [37]. There is also a risk of ‘faux‐design’ research where people with lived experience provide consultation only but do not have any decision‐making input [38]. This study highlighted a difficulty commonly encountered in clinical projects and initiatives, where funding is provided to address problems identified within services, by service providers, with limited pathways and opportunities to slow processes down to ensure meaningful co‐design. We have chosen to write about our difficulties and decisions as an act of transparency and knowledge‐generation about the reality of developing and implementing interventions within clinical settings. Studies that don't go to plan require a ‘rigid flexibility’, defined by CohenMiller et al. [39] as ‘*maintaining a clear and unwavering goal in research with a willingness to be flexible in how it is reached*’ (p. 5). Rigid flexibility allows researchers a way to look at problems in new ways and to reframe ‘failure’ as opportunity. Throughout this phase of the project, while the research team maintained a commitment to ‘doing something’ to support isolated parents within the diverse community across the perinatal period, the ‘failures’ encountered in cohesively identifying and developing the intervention allowed for increased reflective discussion about the ethnocentrism of services, assumptions about community needs and a renewed commitment to interservice, cross‐disciplinary trying. Phase two of this project will focus on the piloting of the group intervention within existing service structures as an attempt to deliver a culturally sensitive group intervention to pregnant women and their families.

## 6 Recommendations for Practice, Policy, Education and Research

Involvement and engagement of parents from culturally diverse backgrounds should occur at the design stage of any proposed intervention to increase the likelihood that the plan will suit the needs of the population. Despite attempts to ensure inclusivity and curiosity into the scope of this project, assumptions of ‘sameness’ or a hierarchy of needs permeated the proposed project before conception and as such, local interventions may be best placed to respond to local needs as they arise rather than focusing on generic risks and vulnerabilities amongst groups, despite good intentions.

This study highlighted the commitment of health service workers and clinicians to providing integrated and responsive care to women who may have decreased engagement with mainstream services, alongside the need for consideration of the deep cultural sensitivities that may underpin disengagement. Careful co‐design with communities may assist in ensuring any perinatal intervention positioned at the interface of mental health, cultural diversity and social isolation is addressing the issues through a culturally sensitive lens.

## Author Contributions

**Sophie Isobel:** conceptualisation, investigation, funding acquisition, writing–original draft, methodology, formal analysis, project administration, supervision, writing–review and editing. **Kahala Dixon:** investigation, writing–review and editing, project administration. **Alison Tutt:** conceptualisation, investigation, funding acquisition, writing–review and editing. **Bridget Clay:** writing–review and editing, investigation. **Sylvia Lim‐Gibson:** conceptualisation, investigation, writing–review and editing, project administration.

## Ethics Statement

The study has ethics approval from the Royal Prince Alfred Hospital Human Research Ethics Committee.

## Conflicts of Interest

The authors declare no conflicts of interest.

## Data Availability

Data from this study is not publicly available.
